# In Vitro Studies for Investigating Creep of Intervertebral Discs under Axial Compression: A Review of Testing Environment and Results

**DOI:** 10.3390/ma15072500

**Published:** 2022-03-28

**Authors:** Mengying Yang, Dingding Xiang, Song Wang, Weiqiang Liu

**Affiliations:** 1 Department of Mechanical Engineering, Tsinghua University, Beijing 100084, China; ymy18@mails.tsinghua.edu.cn; 2 State Key Laboratory of Tribology, Tsinghua University, Beijing 100084, China; xiangdd@mail.neu.edu.cn; 3 Tsinghua Shenzhen International Graduate School, Tsinghua University, Shenzhen 518055, China; 4 Biomechanics and Biotechnology Lab, Research Institute of Tsinghua University in Shenzhen, Shenzhen 518057, China; 5 School of Mechanical Engineering and Automation, Northeastern University, Shenyang 110819, China

**Keywords:** intervertebral disc, creep, in vitro, mechanical testing, biomechanics

## Abstract

Creep responses of intervertebral discs (IVDs) are essential for spinal biomechanics clarification. Yet, there still lacks a well-recognized investigation protocol for this phenomenon. Current work aims at providing researchers with an overview of the in vitro creep tests reported by previous studies, specifically specimen species, testing environment, loading regimes and major results, based on which a preliminary consensus that may guide future creep studies is proposed. Specimens used in creep studies can be simplified as a “bone–disc–bone” structure where three mathematical models can be adopted for describing IVDs’ responses. The preload of 10–50 N for 30 min or three cycles followed by 4 h-creep under constant compression is recommended for ex vivo simulation of physiological condition of long-time sitting or lying. It is worth noticing that species of specimens, environment temperature and humidity all have influences on biomechanical behaviors, and thus are summarized and compared through the literature review. All factors should be carefully set according to a guideline before tests are conducted to urge comparable results across studies. To this end, this review also provides a guideline, as mentioned before, and specific steps that might facilitate the community of biomechanics to obtain more repeatable and comparable results from both natural specimens and novel biomaterials.

## 1. Introduction

In vitro mechanical testing of intervertebral discs (IVDs) provides a valuable method in elucidating the biomechanical properties of natural IVDs, including visco-elasticity and poro-elasticity, which are closely related to disc degeneration and help with the development of bionic IVD prostheses with mechanical mimicry [1]. However, due to the sophistication of natural IVDs structures and physiological conditions, simplifications are needed in animal models, specimens’ preparation, environment setting and loading regime selection. As a result of non-standard selection, different compromises within studies make their results difficult to compare, thus causing repetitive studies. Several studies have proposed standardized test methods for multi-level specimens in bending [2,3], while specifics for in vitro compressive creep investigations are still not available.

In in vitro study, spinal specimens from the human cadavers are the best choices. While due to the scarcity of human specimens, animal models have been used on a large scale as substitutes for in vitro experiments. Appropriate selection of species is of considerable importance when focusing on specific biomechanical problems, while little consensus has been reported on the selection of the species.

Previous studies have demonstrated the influences of disc hydration [4,5,6,7], preconditioning [8,9] and environment settings [1] on testing results, highlighting the importance of consistency in specimen preparation and condition selection. Given that the long-term axial compression is the most common form of load in the spine during everyday activity, it is of prior urgency to set standards of the above factors for in vitro creep tests.

Creep is a time-dependent response of IVD and is a typical feature of viscoelastic materials. In 1982, Twomey et al. [10] defined creep as the progressive deformation of a structure under the constant load when the materials are stressed below their fracture thresholds. One of the most intuitive phenomena, which suggests the formation of creep is that the human body has a height change of 1–2 cm per day [11,12]. To date, several studies described the creep behaviors of the spine under axial compression [13,14,15,16,17,18,19,20,21,22,23,24,25,26]. These investigations highlighted the non-linear and time-dependent behaviors of natural IVDs, showing a rapid decrease in axial height after early compression followed by a slow decrease until reaching equilibrium. Nevertheless, a normalized loading protocol is not yet available to which every study adheres, thus resulting in a significant challenge in comparing mechanical results across studies and hampering the development and testing of biomaterials used for spinal implants [27,28].

With the aim of providing researchers with an overview of the in vitro tests conducted, this study amalgamated the specimen species, testing environment, loading regimens and the major results from previous studies, excluding studies using human specimens. After scanning the titles and abstracts, finally 56 peer-reviewed papers were found to be relevant, of which the earliest was published in 1983. Furthermore, this review provides a standardized approach for in vitro creep testing and advances towards facilitating the community of biomechanics to obtain more repeatable and comparable results from both natural specimens and novel biomaterials.

## 2. Mathematical Models of Creep

Three mathematic models are commonly used for describing the creep behavior of IVDs under compression (i.e., deformation or strain under a constant load), which are the Kohlrausch Williams Watts function (KWW), the Double-Voight model (DV) and the Kelvin three-parameter solid model (Kelvin). The first two equations of displacement (mm) are functions of time (t), while the Kelvin model describes the change of strain (%) with time (t).

(1) $$x\left(t\right)=d_{\infty }\left(1-e^{-{\left(\frac{t}{\tau }\right)}^{\beta }}\right)$$

(2) $$x\left(t\right)=L\left[\frac{1}{S_{1}}\left(1-e^{-\frac{t}{\tau_{1}}}\right)+\frac{1}{S_{2}}\left(1-e^{-\frac{t}{\tau_{2}}}\right)+\frac{1}{S_{E}}\right]$$

(3) $$\varepsilon \left(t\right)=\frac{\delta_{0}}{E_{1}}+\frac{\delta_{0}}{E_{2}}\left(1-e^{-\frac{t}{\tau }}\right)$$

The KWW function (Equation (1)) is a stretched exponential function. The three parameters of this model are the changes of disc height at equilibrium (d_∞_), the time constant (τ) and the stretch parameter (β). A stretched exponential function with β in the interval (0,1] describes an initial response by a faster-than-exponential-decay regime followed by a slower-than-exponential decay regime [29].

The Double-Voight (DV) model (Equation (2)) describes the mechanical behavior of two damper/spring connected in series. The four model parameters are the deformation of the fast Voight model at infinity L/S_1_, the time constant of the fast Voight model τ_1_, the deformation of the slow Voight model at infinity L/S_2_ and the time constant of the slow Voight model τ_2_. Since creep is defined as the deformation under a constant load, these parameters are calculated from the time when the applied load remains constant. Therefore, the L/S_E_, which represents the deformation before the creep phase, was not used [30]. 

The Kelvin three-parameter solid model (Equation (3)) consists of a linear elastic body connected in parallel with an ideal viscous body, then connected with a linear elastic body in series. The three model parameters are the instantaneous elastic modulus at the beginning of creep (E_1_), the elastic modulus of the material in the creep stage (E_2_), and the time constant τ (τ = η/E_2_, η is the viscosity coefficient).

## 3. Factors That Can Influence the Mechanical Properties of IVDs

### 3.1. Species

#### 3.1.1. Difference in Geometry

The geometry of animals and human IVDs varies, and thus should be taken into consideration. Geometric parameters from various animals’ IVDs have been previously measured [31,32,33,34,35], including baboon, sheep, rabbit, rat, mouse and bovine in terms of height, lateral width, anterior–posterior width (AP width) and area. According to the results, human lumbar specimens were larger than all of the aforementioned animal specimens. The value of normalized AP width, which was scaled by the lateral width, was 0.665 for human lumbar discs, and the normalized AP widths for baboon, sheep and mouse lumbar discs were close to human lumbar discs, indicating the similar shapes of those animals’ discs with the human lumbar discs, which were all like ‘kidney bean’.

#### 3.1.2. Difference in Glycosaminoglycan (GAG) and Water Content

The amount and distribution of GAG in IVDs are functionally important to define swelling pressure, water content and compressive properties [36,37,38]. Generally speaking, GAG and water analysis of specific regions indicate a similar trend, featuring higher GAG and water content levels in the nucleus pulpous (NP) and lower amounts of GAG and water in the annulus fibrosus (AF) [39,40,41]. In addition, GAG and water content were significantly different across species. According to results from Jesse et al. [34], GAG contents in NP of IVDs from calf, porcine, sheep, rabbit, rat and cow tail were similar to those of human species (466 ± 205 μg/mg). GAG contents in AF of IVDs from calf, baboon, rabbit, cow tail indicated approximately the same or higher content with AF from human species (269 μg/mg). The water content of calf, porcine, baboon, sheep, rabbit, rat lumbar, cow tail and rat tail IVDs was similar to that of human in the NP and AF, which were 81% and 76% separately.

#### 3.1.3. Difference in Axial Compressive Mechanics

Jesse et al. [34] conducted experiments to acquire the axial compressive mechanical parameters, including stiffness, range of motion (ROM), step and creep displacement using IVDs from various animals under the same loading protocol (cyclic loading followed by creep test under 0.48 MPa for 1 h), and revealed the impact of species related factors (GAG content, water content and size) on IVDs biomechanics. Their study concluded that the compressive stiffness of the baboon (1426 ± 382 N/mm) and sheep lumbar discs (1432 ± 334 N/mm) was closed to that of humans (1734 ± 446 N/mm). The ROM of cow tails (1.24 ± 0.31 mm) was most similar to that of humans (1.21 ± 0.18 mm). The step displacement of cow tail (1.45 ±0.60 mm) was closed to that of humans (0.90 ± 0.12 mm). The creep displacement of porcine (0.55 ± 0.18 mm), baboon (0.36 ± 0.11 mm), sheep (0.24 ± 0.03 mm), rabbit (0.47 ± 0.17 mm), rat lumbar (0.19 ± 0.02 mm), rat tail (0.40 ± 0.11 mm) was similar to that of humans (0.55 ± 0.03 mm). Their study further demonstrated that axial mechanics of IVDs were similar across species, indicating that the values between species were comparable by altering the load magnitude. In experiments simulating disc herniation, sheep IVDs are good choices with a similar shape to human lumbar IVDs and also herniated at the posterolateral region [42,43]. Large animal models (sheep, ovine, pigs) are suitable for investigating effects of mechanical factors, implant preclinical trials and surgical techniques on biomechanics, while small animal models (rat, mouse) are suitable for studying biological processes. When IVD pressure is of focus, animal models with a smaller cross-sectional area than human IVDs can be used, and the applied load needs to be changed proportionately [44,45].

### 3.2. Specimen Harvesting and Storage

**Figure 1:**
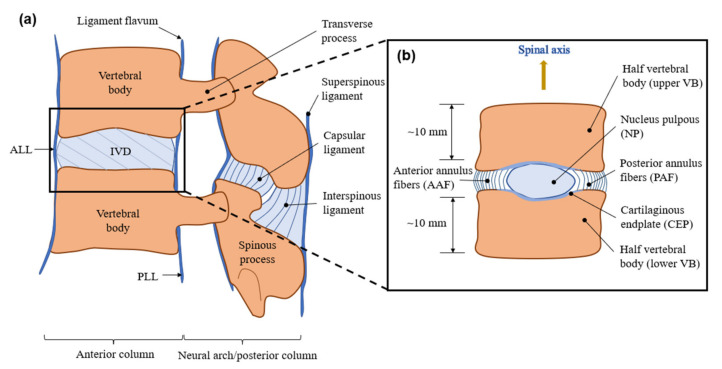
Schematic diagram of (**a**) functional spinal unit (FSU) (**b**) bone–disc–bone structure.

Specimens of in vitro creep tests should be carefully processed into bone–disc–bone structures by making parallel cuts at the upper and lower vertebral bones with a distance of ~10 mm from the IVD using a bone saw under aseptic conditions. The functional segment unit (FSU) is more likely to be used in a kinematic study (Figure 1a). While in biomechanical tests, soft tissue, pedicles and posterior elements should also be removed (Figure 1b), and vertebral bodies can be embedded in polymetheylmethacrylata (PMMA) dental cement to ensure they are paralleled during the loading period. A separate disc without endplates can be used with external restrictions to prevent extrusion.

It should be noted that both vertebral bones and disc creep under prolonged constant loads. Although the creep of vertebral bones is tiny, several studies presented studies quantifying differences with or without bones. Schmidt performed a series of experiments [8,46] to compare the bone–disc–bone structure with the separate disc and demonstrated specific results with regard to displacement, stiffness and pressure under the same condition. The load regime included an 8 h preload under 0.06 MPa uniaxial compression, followed by 10 cycles of 10 min, in which the force was altered between 0.06 MPa and 0.5 MPa. Combined with the research of Oravec et al. [47], three types of specimen structures were used in total as follows: (1) a disc with endplates, (2) a disc without endplates and (3) an isolated vertebral body. The data in Table 1 are collected from the aforementioned studies.

From data in Table 1, the disc contributed to the majority of the height loss during creep, and the bony endplates also exhibited significant impacts on the whole creep value (0.341 ± 0.269 mm under 1000 N after 2 h). Therefore, studies using bone–disc–bone specimens should exclude the height loss of vertebral bodies by appropriate methods of measurement in order to avoid larger and inaccurate results. It can be also seen that the stiffness of specimens with and without endplates was the same (700 N/mm). In the specimen without endplates, the intradiscal pressure was reduced by nearly 50% (~0.43 MPa). This may have contributed to the easy extrusion of the disc without circumferential constraints formed by the annulus and the bony structure, thus resulting in relatively unstable stress and a larger area of the cross-section. It is a reminder that a separate disc is an unsuitable structure in the study related to intradiscal pressure measurement.

Specimens for in vitro creep studies should be stored at −20 °C before testing. McMillan et al. [48] reported that, in case of inappropriate IVD storage (unloaded in a wet environment), swelling by 20% was noted due to the tension in the ligamentum flavum, generating pressure in NP for approximately 70 kPa [49]. Generally, a single freeze–thaw cycle exhibited minimal effects on the intradiscal pressure, stiffness, creep behavior of IVDs [50,51,52] and on the tensile property of AF [53]. However, after several freeze–thaw cycles, significant differences in the joint flexibility may occur [54], and several studies have reported mechanical differences between fresh and frozen IVDs [55,56]. To correct these differences, several studies verified that specimens immersed in saline solution or phosphate-buffered saline for more than 8 h prior to testing may be corrected for physiological hydration status [57].

### 3.3. Testing Environment

During experiments, hydration should be maintained to avoid the effects of dehydration by the following guidelines: (1) testing in a humidity chamber, (2) immersing in 0.15 M phosphate-buffered saline (PBS: 137 mM NaCl, 2.7 mM KCl, 5.4 mM Na_2_HPO_4_, and 0.6 mM KH_2_PO_4_) or saline solution (0.9% or 0.15 mol/L NaCl), or (3) wrapping in saline-soaked gauze [28]. The majority of studies immersed specimens in saline solutions during tests (Table 2), while other studies placed specimens in the custom build culture system, in which the environment was kept at 37 °C with the presence of 5% CO_2_ [58,59], simulating the physiological conditions. To prevent infection, 10,000 u/mL penicillin, 250 mg/L streptomycin and 1.5 mg/mL amphoterizin B should be added in experiments lasting for more than 20 h [2]. A previous study focused on the influence of osmotic pressure on the fluid flow of IVDs with the change in concentration of PEG or NaCl [60]. Those studies conducted tests in the air [61,62] failed to maintain the hydration status, and their results should be treated dialectically.

Temperature is another factor that needs to be controlled. It was reported that body temperature at 37 °C could cause a 10% higher creep under compression than that at room temperature [63], while the results have not been confirmed by other studies.

**Table 2:** Overview of the species, spinal levels, structures and testing environment of in vitro creep studies (under axial compression). The references are listed in chronological order.

Ref.	Number of Samples	Species							Spinal Level				Structure			Testing Environment					
		Porcine	Bovine	Sheep	Canine	Murine	Monkey	Rabbit	Cervical	Thoracic	Lumbar	Coccygeal	IVD Only	VB–disc–VB	FSU	Room Temp.	Body Temp.	Air	Chamber	Saline Bath	Saline Soaked Gauze
[64]	12				☑						☑			☑							☑
[65]	54						☑			☑	☑										
[66]	5			☑							☑				☑		☑		☑	☑	
[67]	21	☑										☑		☑		☑				☑	
[33]	40	☑										☑		☑		☑					☑
[68]	16	☑									☑			☑							☑
[69]	5		☑									☑		☑						☑	
[70]	16		☑									☑		☑		☑				☑	
[71]	10			☑							☑			☑							
[72]	7	☑									☑			☑				☑			
[73]	12		☑									☑		☑				☑			
[74]	43					☑						☑		☑		☑				☑	
[75]	16					☑					☑	☑		☑			☑			☑	
[76]	16	☑									☑			☑			☑			☑	
[37]	60					☑					☑			☑			☑			☑	
[77]	24			☑							☑			☑		☑				☑	
[78]	218	☑							☑						☑	☑					☑
[79]	11					☑						☑		☑		☑				☑	
[80]	126					☑						☑								☑	
[34]	45	☑	☑	☑		☑	☑				☑			☑		☑				☑	
[81]	10		☑									☑	☑						☑		
[82]	36		☑									☑	☑						☑		
[83]	48	☑									☑		☑	☑			☑			☑	
[84]	60					☑					☑	☑		☑						☑	
[85]	30		☑								☑			☑						☑	
[86]	57		☑								☑			☑						☑	
[32]	32	☑								☑	☑		☑								☑
[87]	5							☑			☑				☑				☑		
[35]	48	☑							☑						☑						☑
[88]	42				☑							☑		☑						☑	
[89]	1	☑												☑		☑					☑
[90]	32					☑					☑			☑						☑	
[91]	26					☑						☑				☑				☑	
[92]	6			☑							☑				☑				☑		
[93]	24					☑					☑	☑		☑		☑				☑	
[94]	15			☑						☑	☑			☑		☑					
[95]	15			☑							☑			☑		☑					☑
[96]	24		☑									☑		☑			☑			☑	
[97]	12	☑									☑			☑		☑				☑	
[98]	18					☑						☑		☑		☑				☑	
[99]	3							☑			☑			☑		☑				☑	
[100]	21	☑							☑					☑							☑
[46]	44		☑									☑		☑			☑		☑	☑	
[8]	60		☑									☑	☑	☑			☑		☑	☑	
[60]	25			☑							☑			☑			☑			☑	
[101]	30			☑							☑				☑	☑					☑
[102]	8		☑								☑			☑							
[103]	24	☑								☑				☑					☑		
[104]	54		☑									☑		☑			☑		☑	☑	
[105]	12			☑							☑			☑			☑		☑	☑	
[106]	36			☑							☑			☑				☑			
[107]	48					☑					☑			☑		☑				☑	
[108]	24		☑									☑		☑		☑				☑	
[109]	18			☑							☑				☑	☑			☑	☑	
[110]	6			☑							☑		☑			☑				☑	
[111]	9					☑						☑			☑	☑		☑			

### 3.4. Preload, Load Magnitudes and Duration

#### 3.4.1. Preload

Prior to creep tests, preload is necessary for reaching physiological status and squeezing additional water. Moreover, it can prevent postmortem swelling, which often occurs on cadaveric IVDs [112]. The non-linear property of the IVDs suggests that stiffness can increase if a preload is applied prior to creep [113]. Therefore, the results of the experiments with the necessary preload period can be comparable. Although the duration of preload conditioning differed considerably, it was usually short before displacement equilibrium [45,76,83].

Typical forms of preload are ‘static’ and ‘cyclic’. Generally, although certain studies incorporated thousands of pre-cycles [114], a static preload from 10 N to 50 N or three pre-cycles was sufficient for a consistent response of the IVDs [2].

#### 3.4.2. Load Magnitude and Duration

Firstly, the load magnitude is of importance since disc height and intradiscal pressure are directly related [95,115]. Moreover, the strain in the annulus or bulge of the annulus fibrosus could be affected [116]. The magnitude of compressive force applied to the IVD varies in magnitude with changes in body posture, body weight, muscle activity and external loads [117,118,119]. In a study performed in eight healthy subjects, Nachemson and Morris et al. [120,121,122] demonstrated that the in vivo pressures in NP ranged from 0.091 MPa to 0.539 MPa when lying in prone or supine positions, from 0.46 MPa to 1.33 MPa in a seated position and from 0.5 MPa to 0.87 MPa in a standing position. Wilke et al. [115] reported that the highest pressure in the NP, 2.3 MPa, was recorded in a standing subject who was flexing forwards while simultaneously holding a 20 kg mass. These pressure values can be converted into corresponding load values by multiplying the area of the cross-section, which are ~100 N for L45 human IVDs during bedtime rest [31,46,95,115,123,124] and 750–1200 N during daytime activities. The loads for cervical spines are relatively lower than those for lumbar spines. For bovine tails (e.g., C23, C23), 27.7–209.1 N (0.06–0.28 MPa) and 211.6–373.4 N (~0.5 MPa) were simulated for loads of rest and daily activity [31,46,125]. For sheep lumbar, 40–60 N (~0.45 MPa) and 80–180 N (~1.05 MPa) were equal to nighttime unloading and daytime loading, respectively [45,95,105].

**Figure 2:**
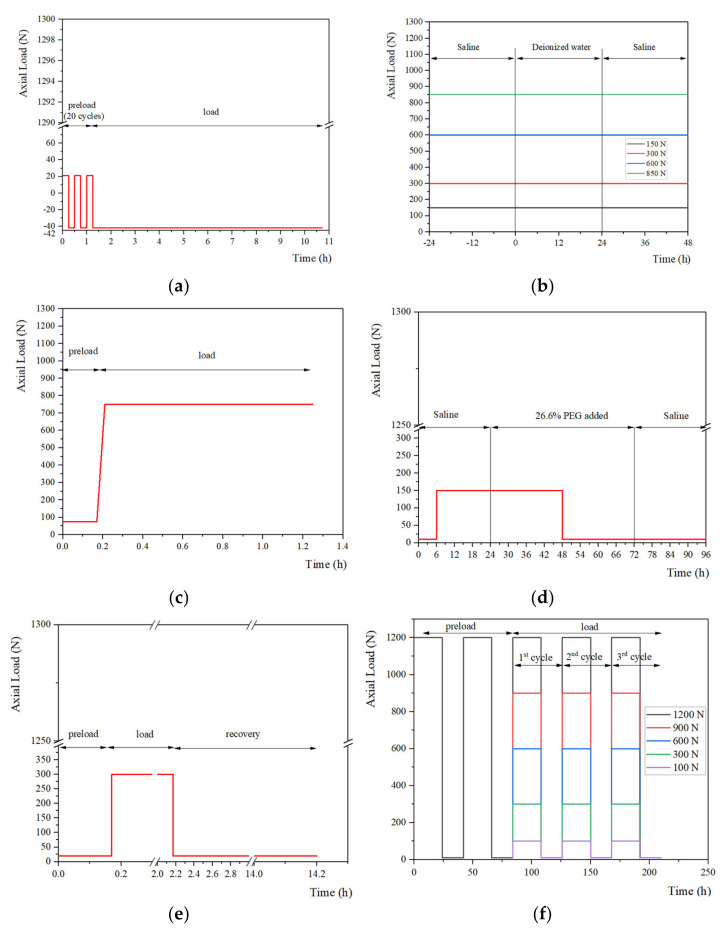
Summary of loading regimes in the static creep experiments. The loading regimes from (**a**) Gullbrand et al. [128] conducted their test with 20 cycles of preload and a static load; (**b**) Vergroesen et al. [60] focused on the effects of the concentration change on creep behavior; (**c**) Hedman et al. [102] adopted both static preload and static load in the creep test; (**d**) Emanuel et al. [129] further studied the effects of changing solution on behaviors of IVDs; (**e**) Bezci et al. [125] paid attention to the height regaining process and the recovery time was longer; (**f**) Bezci et al. [108] conducted tests with static load and unload alternately.

**Figure 3:**
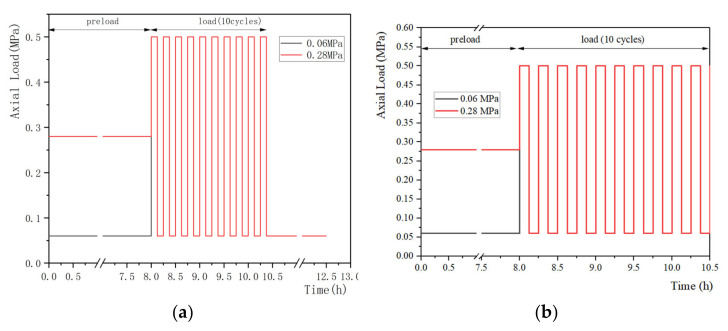
Summary of loading regimes in the quasi-static creep experiments, (**a**) Schmidt et al. [46] and (**b**) Schmidt et al. [8] conducted their test with a static preload and a quasi-static load.

**Figure 4:**
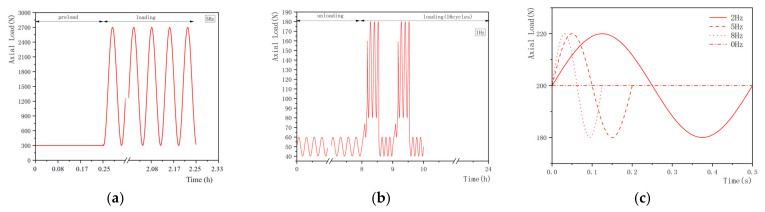
Summary of loading regimes in the dynamic creep experiments. The loading regimes from (**a**) Barrett et al. [100] were a static preload followed by a dynamic load; (**b**) Vergroesen et al. [105] conducted the dynamic test with several cycles of preload; (**c**) Yang et al. [106] conducted the dynamic test without preload.

The representative loading regimes are shown in Figure 2, Figure 3 and Figure 4 and these can be divided into static, quasi-static and dynamic forms. Static load is the most simplified form of daily activities (e.g., sleep and sit) and is widely conducted in studies focusing on the mechanical properties of IVDs during creep. The quasi-static and dynamic loads are used to simulate the human body in physical exercise, driving and other daily activities. In addition, according to the frequencies of the vehicle under normal transportation conditions, the frequencies of vibration can be selected from 0 to 8 Hz in the dynamic creep experiments. Previous studies [126,127] suggested that, due to the resonant frequency of the human body, certain loading frequencies (4–6 Hz) may be harmful. Table 3 summarizes the magnitudes and durations of preloads, loads and the major results from studies.

Secondly, the physiological condition, which is characterized by 8 h-preload and 16 h-load, can be shortened in the in vitro creep study. It has been shown that the measured displacement was more than 80% of the equilibrium displacement following creep for 4 h [57]. However, it should be noted that the creep behavior reached equilibrium within ~12 h [108] and that the time constant of human discs under creep was estimated at 14 h as reported by O’Connell et al. [57].

## 4. Selection of Loading Regime during Creep

### 4.1. Static Load

The static loads from 100 N to 1200 N are usually applied to simulate the diurnal load from the aggregated literature [60,102,108,125,128]. The large range of the selected loads is mainly due to significant differences in the area of cross-section of the specimens used in studies. Moreover, it should also correspond to the physiological conditions of the species according to published in vivo studies. The selection of the duration varied from 10 min to 24 h. Some studies conducted tests for 12–24 h to simulate responses of IVDs diurnally [60,128]; other studies even lasted for several days and were thought to be time-consuming [108]; the reduction to 10 min may be too short to explore the creep behaviors [129]. A previous study indicated that the majority of the equilibrium displacement (>80%) occurred within 4 h [57], and the duration of their creep study was also 4 h, leading to the advice that the duration of 4 h was suitable to reduce the time cost.

### 4.2. Quasi-Static Load

The forces in the quasi-static creep experiments are alternated with high and low loads with a certain interval. The loading regimes in Figure 3 are typical quasi-static patterns, which contain more than 10 cycles of 15 min, including a high load (~0.5 MPa) and a low load (~0.06 or 0.28 MPa) in a cycle. This enables the simulation of the behaviors of the IVDs under loads with frequencies in daily activities. The quasi-static load always lasts for 2–4 h to replicate the in vivo status of the daytime loading period.

### 4.3. Dynamic Load

Dynamic loading regimes can be used to replicate the creep response of IVDs under various physiological frequencies. According to the range of frequency of daily activities (e.g., doing sports or driving), it could be selected from 0 to 8 Hz to simulate the in vivo conditions [106]. In the study from Barrett et al. [100], the mean force of sinusoidal load was 1500 N (Figure 4), which was relatively higher than that of the physiological loads. 130 N and 200 N were used in the studies of Vergroessen et al. [105] and Yang et al. [106] which were considered as rationally based on physiology.

## 5. Techniques for Deformation and Intradiscal Pressure Assessment

### 5.1. Measurements of Deformation

**Figure 5:**
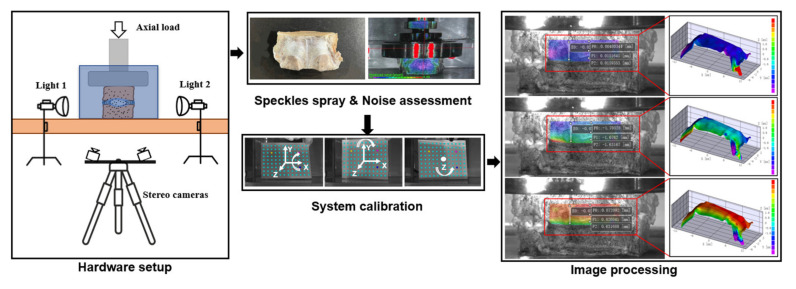
Diagram of the 3D-DIC system for assessing deformation in ex vivo studies. The details of the hardware setup, specimen preconditioning, noise assessment, system calibration and image processing of the system are shown.

Traditionally, creep deformations have been assessed by measuring the disc height change and disc bulging, among which the height loss of IVDs was most investigated. Several mechanical testing devices, such as Instron (model 8872; Instron and IST, Norwood, Canada) and MTS (Mini Bionix 858, MTS Systems Corp., Eden Prairie, MN, USA), are force-controlled or displacement-controlled. With the help of the above devices, axial height changes can be assessed and recorded. While due to the existence of the upper and lower vertebral bodies, the creep value was overestimated by this method as discussed previously, hence calling for novel solutions of the deformation assessment. Inspired by the measurements of the micro strain in the industry of aerospace, digital image correlation (DIC) has been introduced to the biomechanical area for the acquisition of deformation of biological samples. The DIC system is combined with digital cameras with high-resolution (such as A3800; Basler, Exton, PA, USA) and a custom Matlab program or other postprocessing software for texture tracking, which can capture distances of marks placed on two vertebral bodies for accessing strain data [128]. Recent advances in DIC extended this technique to stereovision, designated 3D-DIC, making it possible to perform cross-camera subset matching, in which way the true and three-dimensional positions of each point on a non-planar object can be obtained and calculated. Based on this, the non-contact full-field measurement system (Correlated Solutions Inc., Columbia, SC, USA) was established and could be used in ex vivo studies to yield both axial strains and lateral bulging with high accuracy (Figure 5).

To the best of our knowledge, two factors that required optimization were the shape of the container and the refraction of the water. Firstly, a cubic container was preferred instead of a cylindrical container, since the liquid in the front of the IVD and the arc shape formed a convex lens, amplifying and distorting the tiny deformation. Secondly, the refraction of the water was another interference. Based on the integrated algorithm of the variable ray origin (VRO, Correlated Solutions Inc., Columbia, SC, USA) of the camera model in the 3D-DIC system, this interference could be eliminated. In the VRO camera model, each ray is defined by two points: the first one located in the sensor plane, and the other in a plane parallel to the sensor plane at a distance equal to the focal length along the optical axis. The location of the second point is modeled as a function of the pixel location using polynomial expressions, in which way it enables the 3D-DIC system to eliminate bias when measuring through optical interfaces, such as liquid or container walls.

### 5.2. Measurements of Intradiscal Pressure

**Figure 6:**
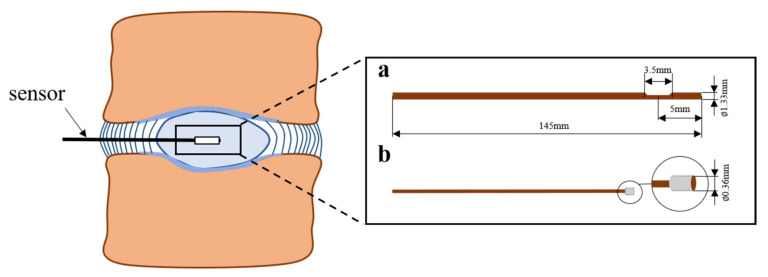
Schematic diagram of needle pressure sensors. Pressure sensors used by Reitmaier et al. [45] and Bashkuev et al. [130].

The widely accepted approach for in vitro measurement of intradiscal pressure is based on needle pressure sensors. The schematic diagram of the sensors is shown in Figure 6.

According to previous studies, information of several models of needle pressure sensors, which are commonly used, are summarized in Table 4.

## 6. Discussion

The current study provides a detailed review of the mathematical models used, the factors that influence the mechanics of the IVDs (species, specimens harvesting and storage, testing environment, preload, load), the studies that attempted to characterize the in vitro creep behaviors of IVDs and the techniques for deformation and intradiscal pressure assessment.

With the continuous development of social aging, diseases concerning IVD degeneration trouble millions of people and adversely affect the quality of life. This trend urges investigations in terms of in vitro creep response with the aim of research and development of IVDs protheses with biomechanical mimicry of natural ones. Unfortunately, due to the lack of standard protocols, the results from different studies are difficult to compare since they are highly protocol-sensitive. Formulation of standards of in vitro creep experiments is urgently required and they should be followed in both natural specimens and biomaterials tested to yield more comparable results.

Firstly, the structure of bone–disc–bone is suitable in ex vivo creep studies. The FSU structure should be abandoned since redundant structures of the spinous process, transverse process and facet joint may share the load during axial compression. Secondly, the testing environment should be set as close as possible to in vivo conditions, notably with regard to the temperature (e.g., 37 °C for the human body, 39 °C for cows) and the osmotic pressure (immersed in PBS or saline). Based on these settings, several studies have established optimal experimental models [8,37,46,60,66,75,76,81,82,83,104,105,109]. Thirdly, the preload of 10–50 N for 30 min or three cycles is appropriate and a data-followed preload can be used for mechanical parameter calculation and mathematical model fitting. Fourthly, it is suggested that the duration of creep should last for 4 h. Longer durations are also acceptable, as long as the decay of specimens is taken into consideration. The addition of 10,000 u/mL penicillin, 250 mg/L streptomycin and 1.5 mg/mL amphoterizin B can protect biological specimens from infection in ex vivo experiments that last for more than 20 h. Moreover, the interference of the deformation of the vertebral bodies on creep measurement must be eliminated, since the existence of vertebras may increase the creep value. Previous studies conducted by Pollintine et al. [13] and Keller et al. [137] that focused on measuring a substantial creep in vertebra are often overlooked. The DIC technique provides an optimal solution for this problem, by which the results of any area or points from the area of interest can be extracted and the data are easily acquired by the postprocessing software, including the 3D displacement and strain.

**Figure 7:**
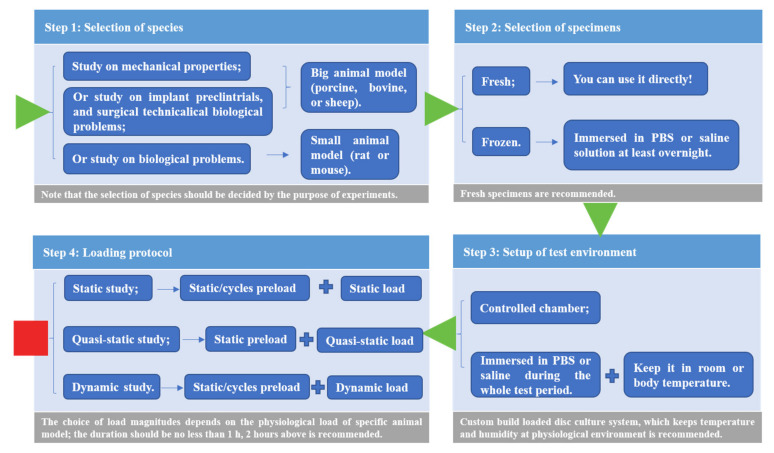
The four-step approach for facilitating in vitro creep experiments.

To obtain more repeatable and comparable results in ex vivo creep studies, the four-step approach can be employed to facilitate experiments conducted in the future (Figure 7).

The approach should follow these steps. Firstly, the selection of species is mainly based on the purpose of the study. Large animal models (porcine, bovine, and sheep) are recommended for mechanical tests, while small animal models (rat and mouse) are suitable for biological studies. Secondly, fresh samples are the best specimens used in ex vivo explorations, while due to their scarcity, frozen samples are acceptable after thawing in PBS overnight. It should be noted that specimens should be abandoned after more than one freeze–thaw cycle. The custom-built culture system is the best choice, while the usual practice for the specimen culture is immersion in PBS or saline during loading. Furthermore, three loading protocols were provided for static, quasi-static and dynamic studies, including the following recommended combinations: (1) static or cycles preload prior to static load; (2) static preload prior to quasi-static load; (3) static or cycles preload prior to dynamic load. The creep responses under the same loading protocol can be compared.

With the development of technology, continuous efforts have been made in terms of characterization of natural IVDs [138], understanding of degeneration-associated alterations [139,140], and development of biomaterials with structure and biomechanics mimicry [141,142,143]. In future treatments of disc diseases, Whatley et al. [144] set high expectations for tissue-engineered IVDs in solving problems that conventional spinal fusion and artificial disc replacement brought. While prior to the development of both conventional and novel constructs, characterizations of mechanical properties of natural IVDs are the first thing that should be considered as their importance for IVD functions. Dynamic and static compression tests are recommended for natural IVDs and implants as the forces on the spine are primarily compressive.

## 7. Conclusions

This work makes efforts towards a consensus that in ex vivo creep investigations, specimens with bone–disc–bone structure are appropriate as simplified models for investigating IVDs’ responses under the preload of 10–50 N for 30 min or three cycles followed by 4-h-creep under constant compression, as well as in the body temperature of corresponding species and immersed in PBS solution for simulating the physiological condition of a long time sitting or lying. In addition, following the four-step approach proposed in this study, it should be believed that future studies will benefit from diminishing challenges in comparing mechanical data across studies. It is apparent that techniques for measuring intradiscal pressure without injury of IVDs are still not fully invented, while, with the introduction of new measurement technology from other areas, certain breakthroughs in biomechanics will emerge without dispute. Future studies may also benefit from non-contact measurements, such as the 3D-DIC technique and laser measurements in the assessment of micro and inner deformation of IVDs. Future works are suggested in the following directions: (1) the influences of novel implants on the transverse distribution of stress in spine and biomechanics of adjacent levels; (2) the responses of IVDs to physiological loading regimes (complex loading modes); (3) the biomaterials with structures that fully mimic the whole IVDs functions anatomically and mechanically. Furthermore, more detailed loading protocols with specific forces and durations are required to be built via combined efforts from the whole community of biomechanics.

## Figures and Tables

**Table 1:** Axial disc height loss, stiffness and maximum pressure were measured following 150 min loading in bovine (1) disc–bone and (2) disc specimens without bony endplates and (3) an isolated vertebral body.

Structure of Specimens	Disc Height Loss (mm)	Stiffness (N/mm)	Maximum Pressure (MPa)	Ref.
Disc with Endplates	1.08	700	1.1	[46]
Disc without Endplates	0.75	700	0.67	[8]
Isolated Vertebral Body	0.341 (±0.269)	1620.75		[47]

**Table 3:** Overview of preload, load and major results from in vitro creep tests. In cases where numerical values were not available, estimates were obtained from the figures. In cases where healthy and degenerate IVDs were tested, the data from the healthy IVDs were recorded. In cases of more than one level of preload or load, the data refer to the highest value. Max. refers to ‘Maximum’. EP refers to ‘Endplate’. The references are listed in chronological order.

Ref.	Preload		Load		Results			
	Static	Cyclic	Static	Quasi-Static	Dynamic	Max. Pressure (MPa)	Max. Displacement (mm) or Strain (%)	Stiffness (N/mm)
[64]	☑		☑				0.7	
[65]			☑				0.528	
[66]	☑				☑		2.0	
[67]	☑		☑		☑		3.5	
[33]			☑				30%	
[68]				☑			1.4	
[69]			☑			3	42%	
[70]			☑	☑			5.2	4000
[71]			☑				2.636	
[72]			☑			0.55		
[73]				☑				
[74]		☑	☑				0.44	
[75]		☑	☑				0.2728 (tail); 0.1728 (lumbar)	12.9
[76]	☑			☑		3.1	3.2	
[37]		☑	☑				0.255	88.3
[77]		☑	☑				0.67 (0.27 creep)	2200
[78]	☑			☑			3	
[79]	☑		☑				0.99 (0.32 creep)	
[80]	☑		☑	☑			0.7 (static); 0.5 (cyclic)	
[34]		☑	☑				0.80 (porcine); 0.73 (baboon); 0.39 (sheep); 0.26 (rat lumbar); 0.11 (mouse lumbar)	2491 (porcine); 1426 (baboon); 1432 ± 334; 78.1 (rat lumbar); 13.0 (mouse lumbar)
[81]		☑			☑		0.53(per day)	
[82]		☑			☑		0.87 (per day)	
[83]	☑			☑			3.1 (VB-disc-VB); 2.6 (only disc)	
[84]		☑	☑				0.34 ± 0.02 (caudal); 0.21 ± 0.06 (lumbar)	
[85]		☑	☑			1.45		
[86]								
[32]	☑		☑					
[87]		☑	☑			1.0		82.7 ± 0.97
[35]	☑		☑		☑		3.6(dynamic); 2.2 (static)	2960 ± 500
[88]	☑		☑			0.51	43 ± 3%	19
[89]	☑		☑				25%	
[90]		☑	☑				0.0996	89 ± 11
[91]		☑	☑				0.4	
[92]		☑	☑					2416 ± 304
[93]		☑		☑			0.0795	
[94]							1.2	
[95]		☑			☑	1.1	0.4	1900
[96]	☑			☑			1.1	
[97]	☑			☑	☑		1.0	1200
[98]				☑			60%	
[99]	☑		☑				1.5	
[100]	☑				☑		2.5	2900
[46]	☑			☑		1.15	5.7 (2.0 creep)	
[8]	☑			☑			1.08 (creep-with EP); 0.75 (creep-without EP)	670 (EP); 690 (without EP)
[60]	☑		☑				2.2	
[101]		☑	☑				0.52 ± 0.14	
[102]	☑		☑			0.46 (AF area)		
[103]	☑			☑		1.7		
[104]	☑			☑		1.1		
[105]		☑			☑		1.2	1900
[106]					☑		47%	
[107]		☑	☑				0.6	85
[108]	☑			☑			7.2	
[109]	☑		☑				0.5 (0.3 creep)	1900
[110]			☑				25%	
[111]	☑		☑				0.16	15

**Table 4:** Summary of needle pressure sensors used in in vitro biomechanical studies.

Manufacturer Name	Model	Species	Segment	Ref.
Gaeltec devices Ltd., Dunvegan, Isle of Skye, Scotland	CTN-4F	Human thoracic and lumbar	T8-T9, L5-S1	[131]
Precision Measurement Company, Ann Arbor, MI, USA	Model 060	Human cervical	C3–C7	[132]
Merit System; Merit Medical Systems, Inc. South Jordan, UT	Not given	Porcine thoracolumbar	L4–L5	[133]
Millar Instruments, Houston, TX, USA	Model SPR-524	Human cervical	C3–T1	[134]
Robert A. Denton, Inc.	Model 6376	Human lumbar	L2–S2	[135]
Shimadzu Corporation	Pinhole pressure sensor	Goat lumbar	T12–S1	[136]
Samba Sensors, Gothenburg, Sweden	360 HP	Bovine tail	C1–C3	[104]
